# On-chip protein separation with single-molecule resolution

**DOI:** 10.1038/s41598-020-72463-z

**Published:** 2020-09-17

**Authors:** Adam Zrehen, Shilo Ohayon, Diana Huttner, Amit Meller

**Affiliations:** Technion Israel Institute of Technology, Haifa, Israel

**Keywords:** Lab-on-a-chip, Single-molecule biophysics, Nanofabrication and nanopatterning

## Abstract

Accurate identification of both abundant and rare proteins hinges on the development of single-protein sensing methods. Given the immense variation in protein expression levels in a cell, separation of proteins by weight would improve protein classification strategies. Upstream separation facilitates sample binning into smaller groups while also preventing sensor overflow, as may be caused by highly abundant proteins in cell lysates or clinical samples. Here, we scale a bulk analysis method for protein separation, sodium dodecyl sulfate–polyacrylamide gel electrophoresis (SDS-PAGE), to the single-molecule level using single-photon sensitive widefield imaging. Single-molecule sensing of the electrokinetically moving proteins is achieved by in situ polymerization of the PAGE in a low-profile fluidic channel having a depth of only ~ 0.6 µm. The polyacrylamide gel restricts the Brownian kinetics of the proteins, while the low-profile channel ensures that they remain in focus during imaging, allowing video-rate monitoring of single-protein migration. Calibration of the device involves separating a set of Atto647N-covalently labeled recombinant proteins in the size range of 14–70 kDa, yielding an exponential dependence of the proteins’ molecular weights on the measured mobilities, as expected. Subsequently, we demonstrate the ability of our fluidic device to separate and image thousands of proteins directly extracted from a human cancer cell line. Using single-particle image analysis methods, we created detailed profiles of the separation kinetics of lysine and cysteine -labeled proteins. Downstream coupling of the device to single-protein identification sensors may provide superior protein classification and improve our ability to analyze complex biological and medical protein samples.

## Introduction

Single-cell proteomics—sensing, identifying, and quantifying the vast number of proteins in a single cell—remains one of the most sought after “holy-grails” in biomedical research. In analogy to the profound impact that single-cell transcriptomics has made in contemporary scientific research, it is broadly anticipated that single-cell proteomics will carry a comparable or larger influence on all aspects of life sciences and medical research^1–7^. Unlike nucleic acids, proteins cannot be replicated in an enzymatic reaction (i.e., polymerase chain reaction). Hence, this limitation, combined with the knowledge that many proteins in a cell’s proteome appear as a single or just a few copies^8^, motivates the development of *single-molecule* sensing techniques. Working towards this goal, single-molecule approaches based on labeled antibodies^9^, Edman degradation^10^, Fluorescence Resonance Energy Transfer (FRET)^11^, and nanopores^12–15^ have recently been developed, but have yet to be applied for profiling an entire proteome. Recent simulation studies of single-protein electro-optical nanopore sensing showed feasibility for whole-proteome identification based on the labeling of only three amino-acids (lysine, cystine, and methionine)^16^. However, the vast dynamic range of protein expression in cells further complicates sensing and analysis^17,18^, and obtaining such high accuracies in practice requires significant improvements in both the spatial and temporal resolutions of single-molecule techniques, the protein labeling efficiency and the development of viable approaches to address the large numbers of protein molecules in each cell.

One strategy that may considerably improve the ability to classify and correctly identify proteins involves the controlled separation of the proteins by their molecular weight, prior to their molecule-by-molecule identification. Not only may this strategy remove highly abundant proteins, such as albumin (representing over 50% of the plasma proteome)^19^, in favor of the more clinically-relevant proteins (e.g., lighter-weight transcription factors, cytokines, etc.), it also potentially improves the accuracy of the identification algorithms, due to the smaller repertoires of proteins that need to be matched in each molecular weight band. For bulk samples, conventional SDS-PAGE has been proven to be a highly efficient method for size separation of proteins. Furthermore, SDS-PAGE has been extended to deal with smaller, albeit bulk, sample volumes using electrophoresis-based microfluidic devices, enabling the quantification of selected denatured proteins^20–28^. A lab-on-a-chip approach is advantageous over conventional SDS-PAGE due to shorter analysis time, reduced sample consumption, and, importantly, better compatibility with downstream single-molecule sensing technologies.

The controlled delivery and separation of proteins at the single-molecule level present a new challenge in terms of both the required photon-sensitive widefield optics and high-resolution microfluidics in which a polyacrylamide gel needs to be embedded. Unlike in bulk SDS-PAGE in which the fluorescent signal is derived from ensembles containing tens of thousands of proteins, the device should enable real-time monitoring of single-protein motion through the fluidic channels. Furthermore, to maintain throughput, hundreds of proteins should be tracked simultaneously, necessitating high-resolution widefield imaging over single-point detectors^29^. Finally, the device should be compatible with downstream sensing approaches, such as single-molecule fluorescence and silicon-based nanopores, which have been shown to be effective at protein detection and classification^30–32^. To meet the above criteria, we developed a complete in-silicon, hard microfluidic device containing a UV-activated polyacrylamide gel for single-molecule separation of fluorescently labeled proteins. The polyacrylamide gel slows down the Brownian kinetics of the proteins, while the low-profile fluidic design (~ 0.6 µm deep) restricts the proteins near the glass surface, permitting high-resolution widefield imaging using high numerical aperture (NA) optics, and real-time imaging of individual proteins during the entire separation process. Calibration of the device is performed by analyzing a set of proteins in the size range of ~ 10–70 kDa (pre-labeled molecular weight), which are shown to separate in less than three minutes. The data empirically agrees with an exponential dependence of the proteins’ molecular weights on their measured mobility in the gel. Following separation, proteins can be ejected from the acrylamide gel into the separating channel for subsequent processing. Subsequently, we demonstrate the ability of our fluidic device to separate and image thousands of proteins directly extracted from a human cancer cell line. Using single particle image analysis methods, we create detailed profiles of the separation kinetics of lysine and cysteine -labeled proteins spanning tens to hundreds of kDa.

## Results and discussion

### Device design and fabrication

**Figure 1:**
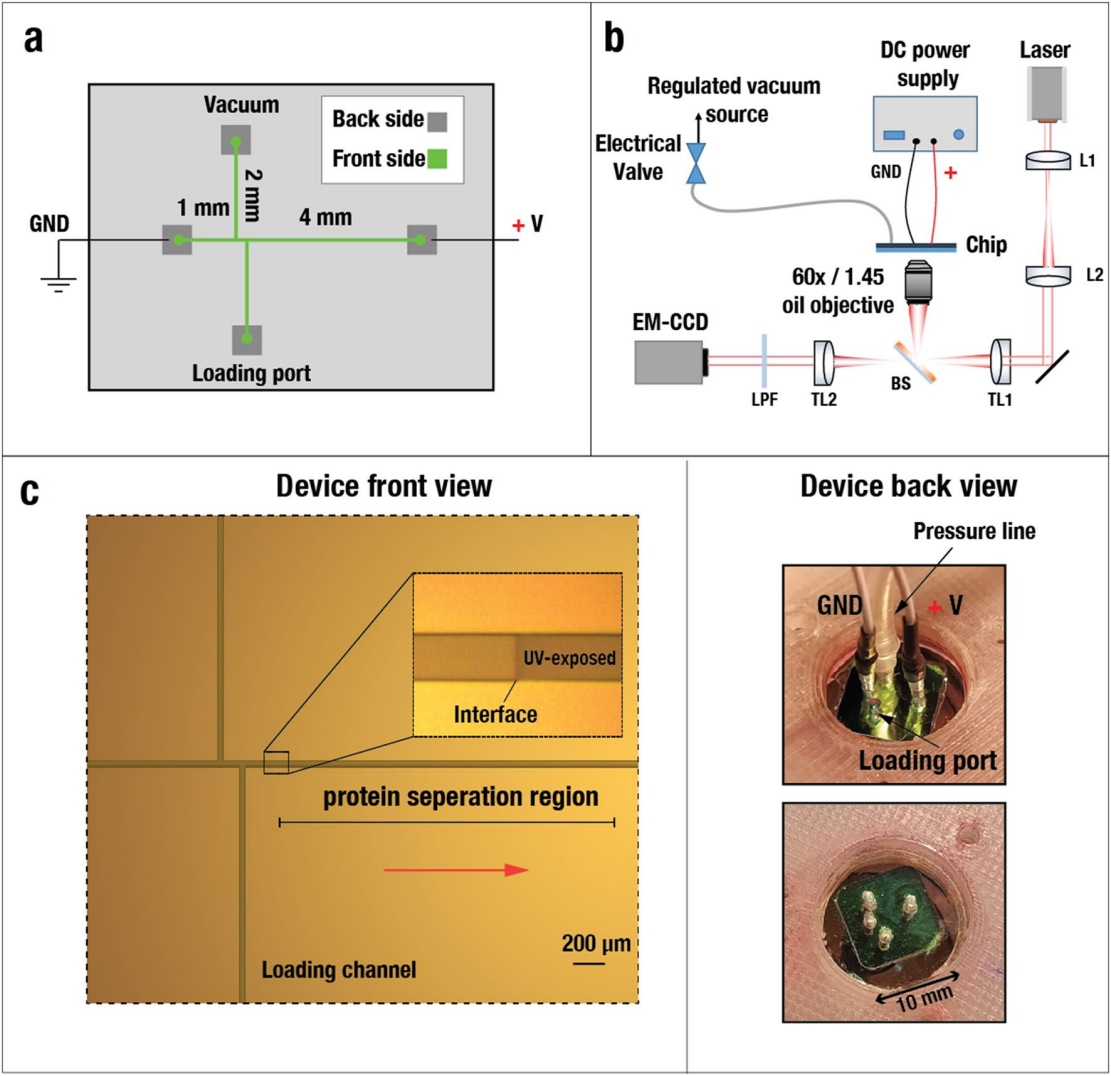
A low-profile silicon-based fluidic device design for protein separation. (**a**) Lithographic pattern for 10 × 10 mm^2^ chip including 50 μm wide microchannels on the front side (green) connected to ports exposed on the backside (grey) and subsequently etched through the thickness of the chip by KOH-etching. The depth of the microchannels is ~ 650 nm. The left and right ports are for connection to ground and positive electrodes, respectively. The top port is for connection to the vacuum line, and the bottom port is for sample loading. The length of the separating channel is 3.6 mm. (**b**) Schematic of setup showing the optical pathway and vacuum and voltage amplifier connections. A 641 nm laser is expanded by lens L1 and L2 and focused onto the chip. The widefield image is filtered by a long pass filter (LPF) and captured on an EM-CCD. TL-tube lens. BS- beamsplitter. (**c**) Left: zoom-in of microchannel design showing offset double T-junction separated by 150 μm center-to-center. The interface of the UV-exposed polyacrylamide gel is further magnified. Right: View of the PDMS layer of the device (back view) to facilitate electrodes and tubing connections.

The on-chip SDS-PAGE design consists of offset double T-junctions as injection arms to the separating channel (Fig. 1a)^23,33,34^. Adjustments to the microchannel dimensions were made to accommodate single-molecule sensing capabilities throughout the separation process. Importantly, the channel depth was made only ~ 650 nm deep to ensure single proteins remain in the field-of-view of a high magnification microscope objective. The low-profile channel also significantly reduces the probability of having more than one protein at each diffraction-limited spot at the same time. The microchannels are connected to four liquid reservoirs/ports by through-holes etched through the silicon substrate on the backside. A single port accepts plastic tubing for applying negative pressure (~ 10 mbar vacuum), controlled by an electrical valve, to load protein molecules. An additional two ports connect to platinum electrodes for applying voltages up to 30 V across the 5 mm channels. A fourth port is for loading protein samples (∼ 1 μL in volume). Glass sealing by anodic bonding, as well as backside PDMS bonding to create sample reservoirs and an interface for tubing and electrodes (Fig. 1b), were performed as described previously^35^. For imaging, we constructed a widefield microscope consisting of an expanded 641 nm laser (iFlex-2000) through a high NA 1.45, 60× oil objective (Olympus PlanApo), as illustrated in Fig. 1b. Images were acquired on an EM-CCD camera (Andor iXon 887) at 19.31 frames per second, and 1 × 1 binning to yield a ~ 267 nm pixel size.

The microchannels were prepared for UV-activated polyacrylamide gel polymerization using a two-step channel coating procedure^36^. Microchannels were first functionalized using acrylate-terminated self-assembled monomers and then coated with linear polyacrylamide in order to minimize non-specific sticking of proteins to the channel walls as described in “Methods”—“Channel coating”. Following surface preparation, 5–10% polyacrylamide gel matrices were prepared by adjusting the total of 40% acrylamide/bis-acrylamide solution with 0.1% (w/v) tris–glycine (0.025 M Tris base and 0.194 M glycine pH 8.3), 0.2% (w/v) SDS and 2,2′-azobis[2-methyl-*N*-(2-hydroxyethyl)propionamide] (VA-086) photoinitiator. The channels were then filled with the unpolymerized acrylamide solution using positive pressure. To induce selective photopolymerization of the gel only in the “separating channel region” right of the T-junction (see Fig. 1c and Supplementary Fig. S1), we took advantage of a recent digital lithography technology (MicroWriter ML3 from DMO, UK), which includes a 365 nm light source producing sufficiently intense illumination to cure the gel. As can be seen in Fig. 1c, the post-cure region appears slightly darker than the unexposed gel. An additional several hundred micrometers of microchannel length was left unexposed at the end of the separating channel so that single proteins could be ejected from the gel. The total cured acrylamide gel length was either ~ 1.5 mm or ~ 2.5 mm, depending on the required separation resolution.

### Protein loading and stacking

Standard gel slab SDS-PAGE experiments involve a stacking gel upstream of the separation gel to concentrate proteins. This is achieved by controlling the charge state of glycine added to the loading buffer by lowering the pH of the gel, causing a steep voltage gradient between the slow-moving glycine front and fast-moving Tris-HCl^37^. A stacking gel is normally needed because the sample reservoir is millimeters in depth. In contrast, our device is designed for a small sample plug size (< 200 μm from end-to-end), which can traverse this distance in less than 2 s, significantly faster than the migration velocity through the whole gel. This obviates the need for a stacking gel. Proteins are subsequently observed to concentrate in a narrow band (~ 8 µm) of the gel.

Another critical design feature involves the configuration of the electric field at the double-T-junction in order to prevent leakage of proteins from the loading sample into the separation channel once the voltage is applied. Silicon substrates leak current at the electrical field intensities used with our device. Hence the positive potential difference applied at the separation channel outlet is partially passed to the loading port, causing proteins present in the loading port to be pulled backward away from the separation channel. The voltage gradient towards the loading port sharply declines at the separation channel junction, and the bulk of proteins in the separation channel are pulled into the gel. Therefore, the sample plug size entering the separation channel is well-controlled (~ 200 μm from end-to-end). This design feature is simpler than that previously implemented, relying on a high fluidic resistance polymer matrix in the loading channel to prevent leakage^23^.

**Figure 2:**
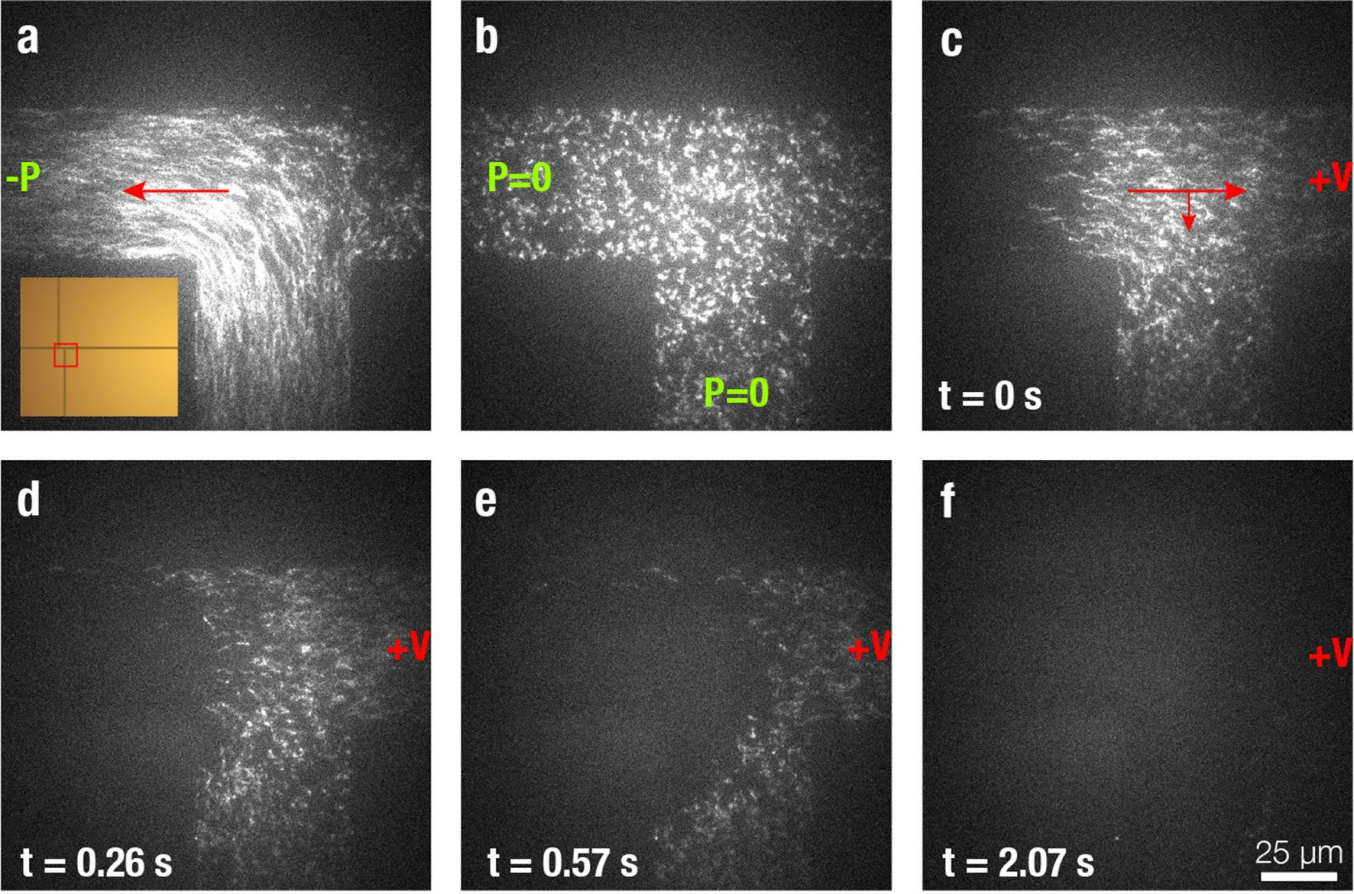
Protein loading in channel. (**a**) The vacuum is turned on, pulling samples from the loading reservoir into the T-junction. Inlet shows the position on the chip. (**b**) The vacuum is turned off, and individual Atto647N-BSA appear distorted due to Brownian motion. (**c**) Subsequently, the voltage (30 V) is turned on, and the proteins are driven in the direction of the electric field. (**d**) The protein plug divides between the separation channel and loading channel, and (**e**) proteins in the loading channel return to the loading chamber. As the electric field is stronger in the separation channel, most proteins present there are driven to the polyacrylamide gel. Similarly, the electric field in the loading channel is biased towards the loading port, preventing protein leakage during separation. (**f**) The proteins disappear from the field-of-view.

Figure 2 and Supplementary Video S1 show a sample experiment in which Atto647N-labeled BSA proteins are loaded into the device and subsequently pulled through the separation channel. The first frame (a) shows the loading phase during which proteins are pulled into the T-junction by negative pressure (~ 10 mbar). Once the T-junction is filled with proteins, the vacuum is turned off, stopping the flow of proteins (b). At this point, individual proteins are already identifiable but somewhat blurred due to their fast Brownian motion. Next, a 60 V/cm potential is applied across the separation channel, resulting in the electrophoretic migration of proteins through the protein channel and towards the loading port, as described above. Over the next few frames (c–f), or approximately 2 s, the proteins disappear from the field-of-view in the direction of the electric field.

**Figure 3:**
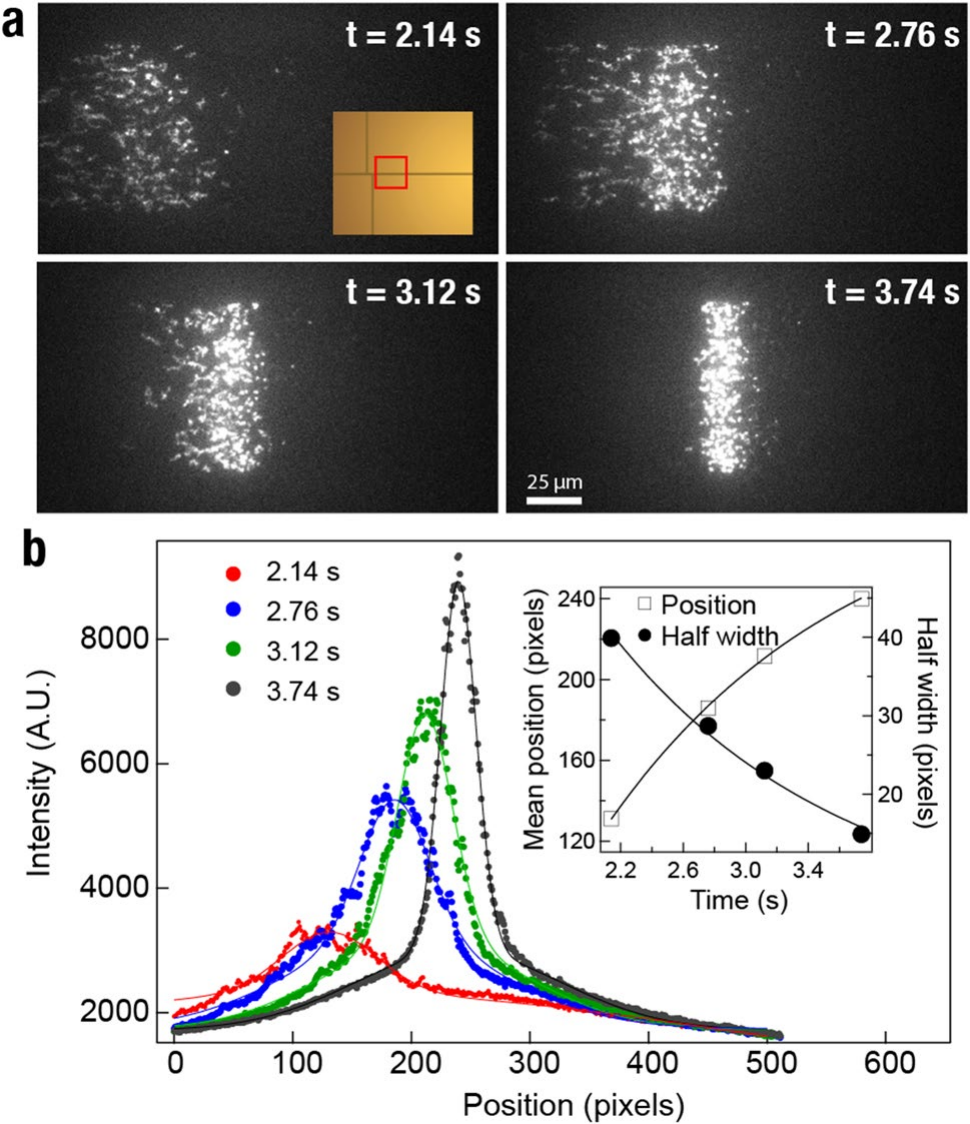
Protein self-stacking at the gel interface. (**a**) Atto647N-labeled BSA proteins are driven towards the polyacrylamide gel (exposed at 12,500 mJ/cm^2^) at a 30 V applied bias. Inlet shows the position on the chip. (**b**) The initial plug with an end-to-end length of ~ 200 µm is concentrated to a width of ~ 8 µm at the gel interface by t = 3.74 s and fitted by Gaussian distributions. Inlet shows the mean position of the fitting and half-width as a function of time.

Figure 3 and Supplementary Video S2 show the protein stacking at the gel interface in real-time for an experiment with Atto647N-labeled BSA proteins at an applied 60 V/cm potential difference. At *t* = 2.14 s, the proteins begin to arrive at the gel interface, and their electrophoretic migration slows down due to increased resistance to movement. In the gel, Brownian motion is strongly dampened, and individual proteins are clearly identifiable. Over the next ~ 1.5 s, all the proteins enter the gel and accumulate into a narrow band ~ 8 µm in thickness, as measured by a Gaussian fitting, performed for each frame (Fig. 3b). The four fittings for each frame capture the principle of protein stacking, as the half-width of the distribution narrows to a minimum as the mean position of the proteins increases. Once attaining this minimum (i.e., a stacked configuration), the protein plug will subsequently expand along the length of the gel as slight differences in protein labeling efficiency result in different migration speeds. Another example using a higher concentration gel is given in Supplementary Fig. S3, demonstrating slower stacking.

### Protein separation

The degree of separation gel crosslinking is affected by the percentage of acrylamide/bis-acrylamide, the quantity of photoinitiator, and the UV-exposure dose^38^. Various conditions of gel percentage and exposure time were tested, with a suitable separation obtained using an 8% gel exposed to an energy density dose of 12,500 mJ/cm^2^ in roughly 5 min. Gel slab SDS-PAGE experiments of individual protein samples of either bovine serum albumin (BSA), carbonic anhydrase (CA), ovalbumin (OA), or lysozyme (LYS) were performed in order to assess the relative protein migration times following covalent labeling. Notably, lysine labeling can add substantially to the bare protein weight^39^, as shown in Supplementary Fig. S2 in the slab gel electrophoresis experiment. For example, a 50% labeling efficiency of BSA would increase its gross molecular weight from 66.4 to 85 kDa (BSA has 59 lysine residues), using a $\Delta$m = 0.6289 kDa per NHS-ester Atto647N conjugate^40^. The increased molecular weight provides an explanation to their significant observed retarded mobility in the gel as compared with that of the unlabeled proteins (Supplementary Fig. S2).

**Figure 4:**
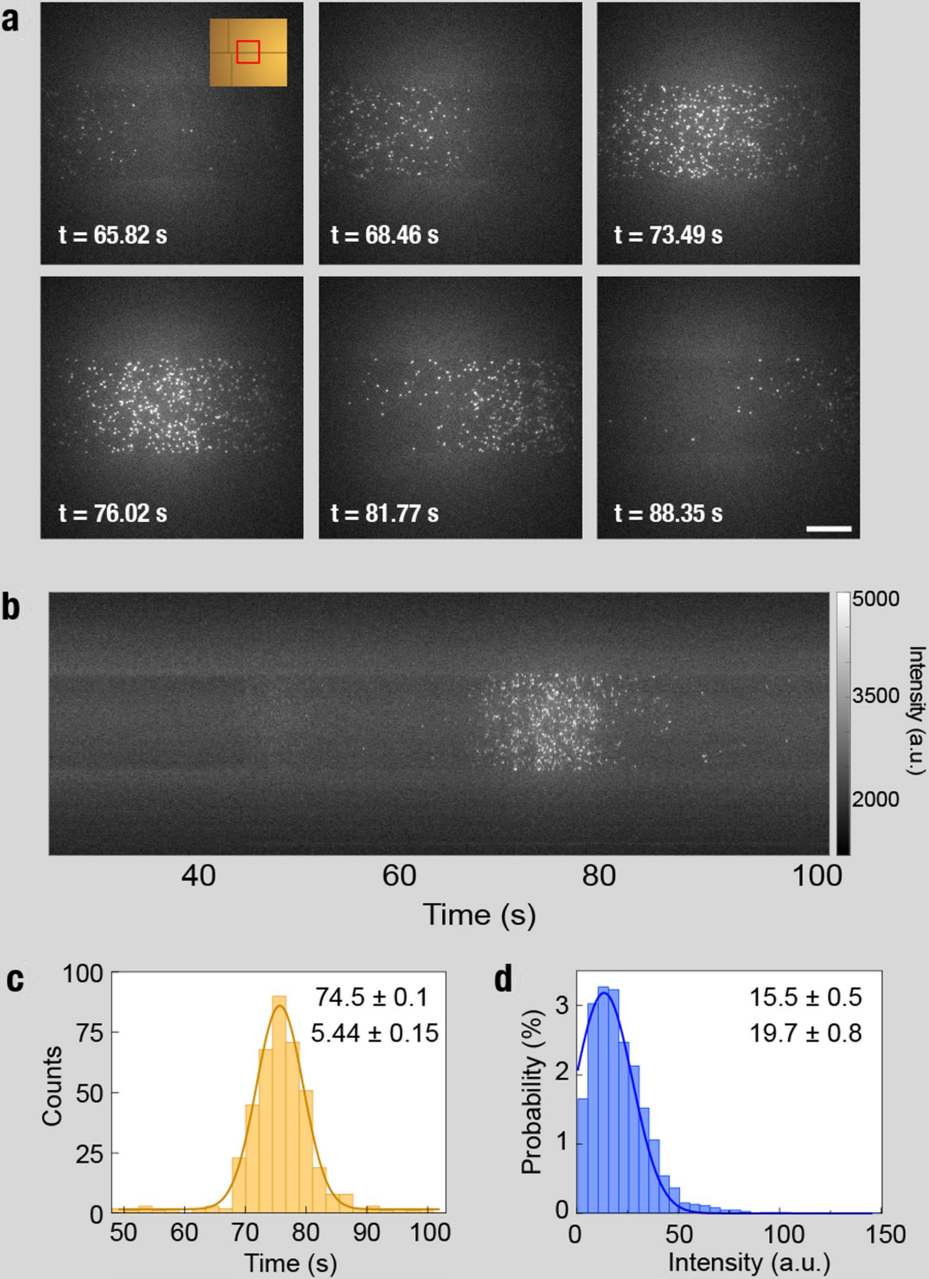
Carbonic anhydrase on-chip migration. (**a**) Single carbonic anhydrase Atto647N-labeled proteins electrically migrating through the channel from left to right under an applied bias of 30 V, imaged 1.3 mm into the channel. Inlet shows the position on the chip. (**b**) Kymograph, formed by concatenating the central pixel column of each frame from a fixed location along the channel (see Methods for kymograph construction), showing a clear protein band forming over time. (**c**) The protein band intensity histogram follows a Gaussian profile with a mean time of 74.5 s and a standard deviation of 5.44 s. (**d**) The normalized fluorescence intensity histogram is fit to a Gaussian probability function with a mean of 15.5 and a width of 19.7 (a.u.). Mean values and full width values and errors are noted.

Electrophoresis experiments of a single species of recombinant purified protein samples were conducted on-chip (1.5 mm gel length). Figure 4 and Supplementary Video S3 show results obtained for CA, in which single fluorescently labeled proteins are differentiable as they move in the direction of the electric field. The top 6 panels in Fig. 4a show snapshots of a specific area in the channel (see inset of the first panel, 1.3 mm into the gel) at different time points. Kymographs were constructed by extracting from each frame the middle column and stacking them along the horizontal axis (Fig. 4b top panel). A faint band at around t = 47 s represents the free Atto647N dyes, and the main protein band appears at t = 74.5 s. A minority of proteins (typically < 1%) non-specifically adsorb to the surface.

We applied a single particle identification algorithm to determine the distribution of protein positions and intensities (see “Methods”—“Protein detection algorithm”). A histogram of the particles’ position fits well with a Gaussian distribution as expected based on bulk studies^37^ (Fig. 4c). Importantly, a normalized histogram analysis of the particles’ intensities, defined as the sum of pixel counts for each particle corrected by the image background, also fits a single Gaussian probability distribution (Fig. 4d), suggesting that the identified particles in our data represent, by large, single-proteins as opposed to multiple proteins, which would have yielded a bimodal distribution. The cut-off at the low-intensity side is due to the imaging background noise. The migration bandwidth (Fig. 4c) is affected by both the stochastic spread of the proteins in the gel and the fact that labeling efficiency can vary among proteins affecting both their mass and electrical charge. In contrast, Fig. 4d, represents primarily the variance in labeling efficiency.

**Figure 5:**
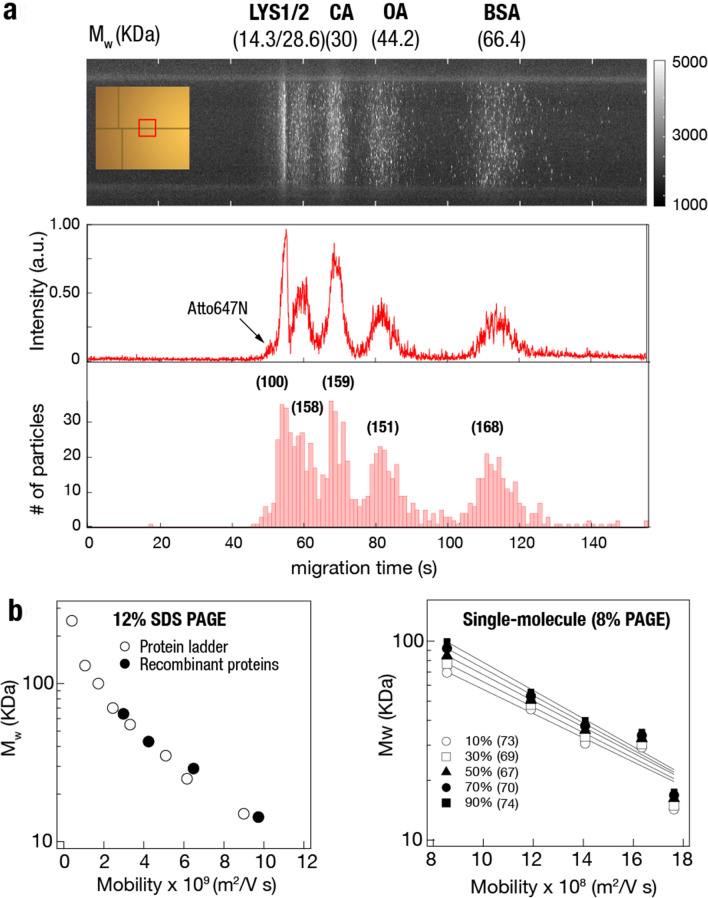
Comparison of protein migration times for differently sized purified proteins. (**a**) Top: Kymograph showing discrete protein bands for the monomers and dimers of LYS, followed by CA, OA, and BSA (see Methods for kymograph construction). The bare molecular weight plus fluorescent labeling weight (assuming 50% labeling efficiency) is given in brackets. Inlet shows the position on the chip. Bottom: Normalized fluorescent intensity measured as the fraction of pixels in a column above a certain threshold, versus protein migration time. Five peaks associated with the five protein populations are visible from the averaged intensity trace (middle panel), with an additional small peak representing the free fluorophores as indicated by the arrow. Lower panel displays a single particle analysis applied to the movie frames, allowing digital counting of number of proteins per peak. (**b**) Left panel: semi-log plot of the molecular weight of unlabeled proteins as a function of measured mobility analyzed in SDS-PAGE (Supplementary Fig. S2). Right panel: single-molecule migration analysis of Atto647N-labeled recombinant proteins shown in (**a**) assuming different levels of labeling efficiencies as indicated. Lines represent exponential fits to the data (fit χ^2^ values shown in parentheses).

Next, using a longer channel of 2.5 mm, we performed separation experiments of a nearly equal molar mixture of purified proteins samples (LYS, CA, OA, and BSA) at 30 V. Our results, presented in the kymograph in Fig. 5a, show well-defined peaks for each of the proteins as indicated at *t* = 55, 69, 82, and 114 s, including an additional peak at *t* = 60 s, attributed to the possibility of LYS dimers. A separate experiment with only LYS proteins confirmed the presence of two protein peaks, representing monomer and dimer populations, as shown in Supplementary Fig. S4, which is consistent with the slab gel electrophoresis result and other bulk studies^41^. Interestingly, LYS dimers migrate slightly slower than half the monomer speed, possibly caused by close interactions between individual LYS monomers resulting in a larger cross-sectional area and greater resistance in the gel. A minor peak at *t* = 50.6 s is attributed to the free Atto647N dyes.

The ensemble averaged line traces extracted from the kymograph (Fig. 5a middle panel) could be precisely reproduced by applying single particle finding analysis to our movie frames (see Methods). Our results (Fig. 5a lower panel) are presented as single-particle histograms as a function of the migration time in the channel. Notably each and every peak in the averaged trace (middle panel) is reproduced in the single-molecule analysis, but importantly this analysis allows digital counting of each of the five mass-separated proteins, as indicated in parentheses above each peak, and relative quantification of the proteins in the sample.

Conventionally, SDS-PAGE protein separations are presented in semi-log plots of the molecular weight. To compare our fluidic microchannel migration profiles with standard bulk SDS-PAGE, we calculated the protein mobilities in the two methods. For the bulk gel analysis, we measured the relative migration distance of each protein band and divided it by the total migration time and the electrical field, using data from Supplementary Fig. S2. For the single-protein microfluidic device analysis, we measured the migration time of each of the protein peaks relative to the free atto647N dye using a travel distance of 2.3 mm and an electrical field strength of 12 kV/m. Our results for the bulk and single-molecule measurements are shown in Fig. 5b. The bulk gel mobilities are roughly one order of magnitude smaller than the device mobilities, attributed to the difference in gel percentage. As the exact labeling efficiencies of the proteins were not known *a-priori*, we evaluated the single-molecule results under a broad range of possible efficiencies ranging from 10 to 90%, and the data was fitted by decaying exponential functions.

### On-chip separation of cell extracts

**Figure 6:**
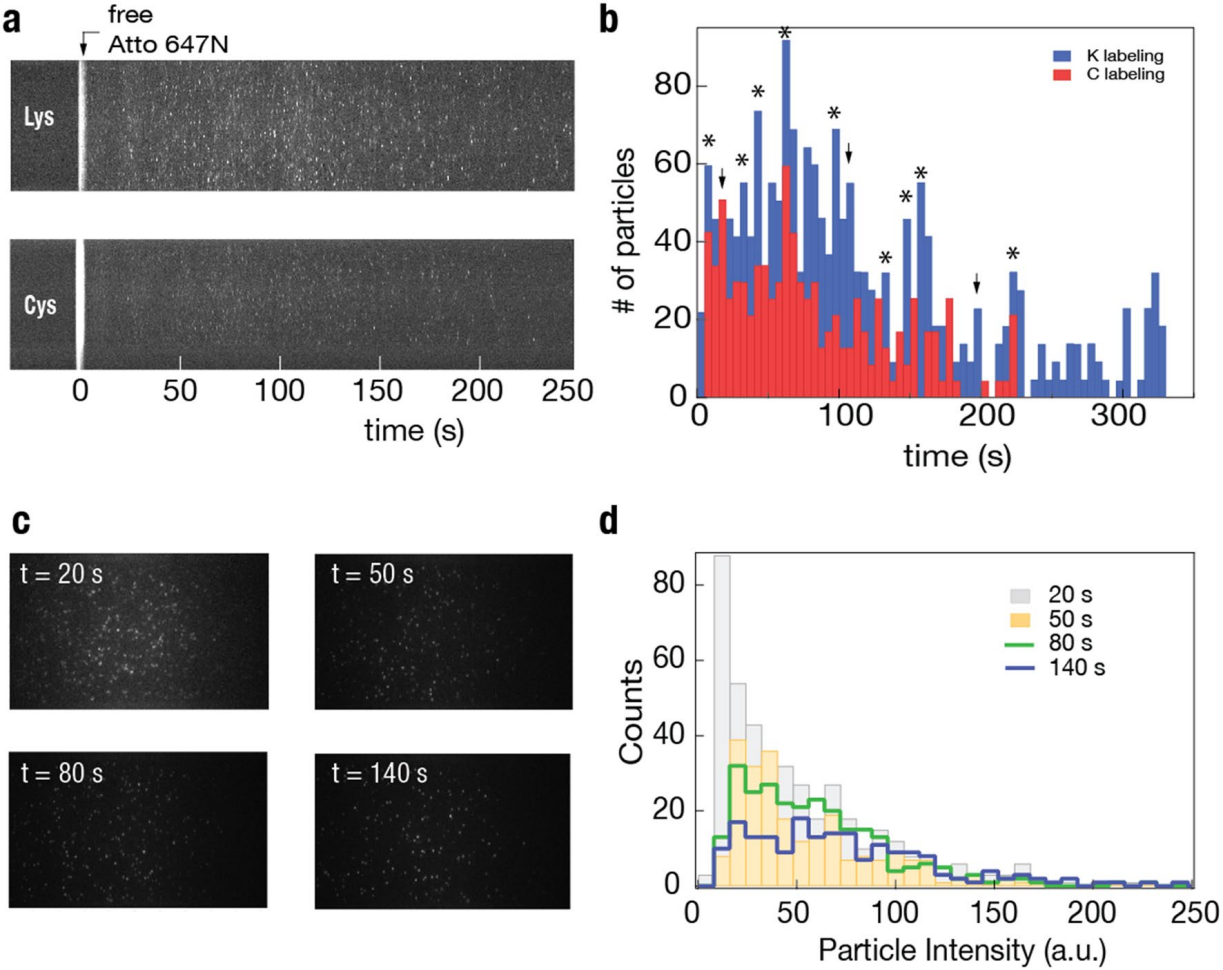
Whole cell extract separations. Proteins extracted from HCT116 colon cancer cells were labeled at the lysine or cysteine residues with Atto647N and separated at 60 V/cm. (**a**) Kymographs for lysine-labeled (top) and cysteine-labeled (bottom) separation experiments where t = 0 s corresponds to the end of the free fluorophore front. (**b**) Single-protein identification histograms as a function of migration time based on the full frame stack analyses. Histograms are binned at 5 s intervals. Asterisks highlight peaks that appear in both the lysine-labeled sample (blue) and the cysteine-labeled sample (red), with a slight delay of the lysine labeling presumably due to their larger molecular weight shift. Arrows refer to peaks that appear differentially in either run. (**c**) Representative video frames correspond to 20, 50, 80, and 140 s for the lysine-labeled experiment. (**d**) Histogram of the proteins’ intensity at different time points, showing a rightwards shift at later timepoints indicating a shift towards heavier but fewer proteins with larger numbers of labeled lysine residues at the longer times.

Moving beyond purified recombinant proteins, we evaluated the ability of our device to separate more complex samples and to bring us one step closer to single-cell proteome analysis. To this end, we labeled cancer cell lysate (as described in the Supplementary Information) with atto647N NHS ester for lysine labeling or with atto647N maleimide for cysteine labeling and used highly diluted samples for on-chip analysis. Figure 6a shows typical protein migration kymographs of the lysine-labeled (top) and the cysteine-labeled (bottom) cell extracts (two additional repeats are provided in the Supplementary Fig. S7). Since the kymographs only represent a small fraction of the entire data (the center column of pixels), in order to maximize the number of extracted protein trajectories, we used the entire frame stacks in the acquired videos, typically consisting of thousands of frames each, and applied the single-particle analysis to obtain the particle counts and intensities. Nevertheless, to avoid the risk of counting some proteins several times, we calculated the typical migration distance per frame and used only non-overlapping regions in successive frames. The non-normalized protein counting histograms measured for the two labeling schemes are presented in Fig. 6b, lysine labeling in blue (1,833 proteins), and cysteine labeling in red (978 proteins).

The cell extract histograms exhibit multiple local peaks at the different migration times (measured relative to the free dye), with the most prominent peak clearly visible roughly at *t* = 60 s. Bulk SDS-PAGE analysis of the same samples (performed at much larger concentrations) confirms the presence of several discrete bands, both when labeled non-specifically using Coomassie blue stain or by the covalent coupling of Atto647N to either lysines or cysteines (Supplementary Fig. S6). The most prominent bands observed in the bulk analysis lie in the molecular weight range of 50–70 kDa, in qualitative agreement with the theoretical proteome analysis of the molecular weight distribution (Supplementary Fig. S5)^42^. Moreover, using our device calibration (Fig. 5), we note that that the main peak in our single-molecule analysis corresponds to proteins residing in a comparable molecular weight range to the bulk SDS-PAGE and the theoretical analysis. Interestingly, several prominent peaks (marked by asterisks) in the lysine and cysteine labeled proteins shown in Fig. 6b appear at nearly the same migration time, with a slight shift towards heavier proteins (longer migration time) of the lysine-labeled proteins, presumably due to the higher abundance of the lysines that results in larger post-labeling molecular weight as compared to cysteine. However, some peaks (marked by arrows) in either of the two runs appear to be differentially labeled and separated.

The qualitative resemblance between the lysine and cysteine-labeled cell extract profiles is encouraging. Yet, it highlights the need to further expand this analysis beyond the current proof-of-principle. We also note that the overall shape of the measured distributions resembles the database-predicted proteome profile displayed in Supplementary Fig. S5. However, the latter does not consider the proteins’ abundancy, the molecular weight shifts or possibly the altered net charge caused by their labeling. Nevertheless, it is important to emphasize that our goal here was to show the feasibility of whole-cell SDS denatured protein extract separation as a way to facilitate downstream single-molecule analysis. Unlike for slab gel electrophoresis, a single-particle analysis may be used to extract the distribution of labeling intensities for each time point in the migration process. An example of one of the lysine datasets is shown in Fig. 6c, where four representative images obtained at *t* = 20, 50, 80, 140 s are presented. The intensity histograms of these frames are displayed in Fig. 6d. To compare similarly among the frames, we normalized the intensity to the maximum level in each frame. As expected, we see a shift in the distribution of particle intensities as a function of migration time. In other words, as time progresses beyond the peak of the distribution (~ 60 s, Fig. 6b), we gradually observe smaller numbers of proteins, and their intensity is shifted to higher values. This analysis further strengthens the conclusion that whole cell’s extract separation and analysis are made possible in our device.

### Protein ejection

**Figure 7:**
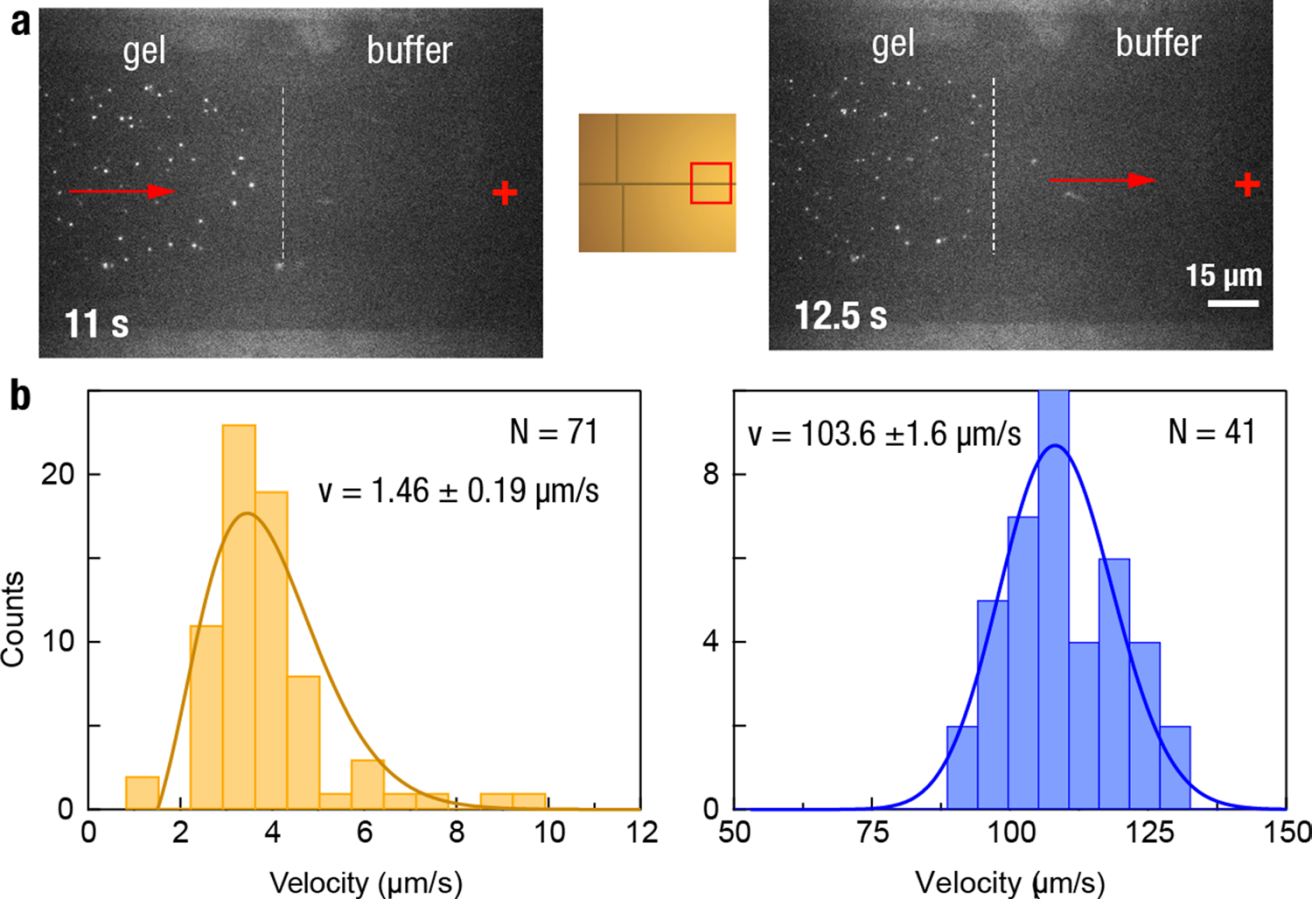
Characterization of single protein velocity distributions before and after exiting the gel. (**a**) Individual gel-embedded Atto647N-labeled BSA proteins are pulled in the direction of a 30 V applied bias until they reach the gel exit (indicated by the white dashed line) and accelerate from a mean of 1.46 ± 0.19 µm/s to 104.5 ± 1.3 µm/s. Inlet shows the position on the chip. (**b**) Histogram of protein velocity before (yellow, N = 71 samples) and after (blue, N = 41 samples) exiting the gel. Notice the different scales of the x-axis in each graph.

For on-chip single-molecule protein separation to be compatible with downstream sensors such as nanopores, the protein should be able to escape the polyacrylamide gel after separation. Moreover, proteins of different sizes may need to be trapped in the gel until sensing is required if downstream processivity is limiting. In Fig. 7 and Supplementary Video S4, the principle of single-protein trapping and release is demonstrated: Individual BSA proteins, initially trapped in the gel, are subsequently driven outside of the gel by applying a positive voltage potential. As shown in Fig. 7b, the migration speed within the gel is uniform, as expected for a single protein population. It subsequently increases ~ 70 × in the low resistance channel. In principle, protein populations above a certain molecular weight can be trapped either temporarily or permanently in the gel to prevent overflowing a downstream sensor. Alternatively, the lower molecular weight proteins exiting the chamber first can be sent to a waste stream, or in a more advanced configuration, different protein populations mixed within the separation signal can be assigned to different downstream locations.

## Conclusions

Herein, we presented a simple-to-fabricate in-silicon device and optical platform for single-molecule protein separation, visualization, and counting. Electrophoretic separation of proteins in the polyacrylamide-embedded low-profile fluidic channel allowed single-particle tracking using high-sensitivity widefield fluorescence imaging. Calibration of the device with fluorescently labeled proteins of known molecular weights resulted in migration mobility values in line with the measured values using bulk SDS, allowing us to relate relative migration time with the mass of the labeled proteins. Single-particle detection applied to our movie frames (Fig. 5a) permits counting of the mass-separated particles in the sub-micron channel. This analysis allows relative quantification of different proteins in mixtures with a digital accuracy. To further demonstrate the ability of our device to separate a broad range of protein masses from biological sources, whole-cell extract separations using two different labeling schemes were executed on-chip (Fig. 6), producing characteristic profiles, which were consistent with each other and in qualitative agreement with the theoretical proteome distribution analysis (Supplementary Fig. S5). However, the high complexity of the proteome and the limited statistics complicate the interpretation of the single-particle histograms and may benefit from future developments of pattern recognition methods.

Much work remains to increase the throughput of the system and move closer to the goal of sampling the entire proteome in a single device. Moving towards single-cell proteome analysis requires integration with upstream methods capable of single-cell trapping and lysis^43–46^, as well as fluorophore labeling. Additionally, superior protein tracking algorithms may allow continuous protein separation without stacking by assigning protein mobilities in a single field-of-view. Future studies may involve repeated cell extraction analyses and single-cell proteome extraction and separations^47,48^ for studying cell-to-cell variability, or integration with single-molecule sensors such as solid-state nanopores.

## Methods

### Protein labeling

Recombinant proteins (Sigma Aldrich) were incubated at 37 °C for 0.5–1 h in freshly prepared and degassed reaction buffer, consisting of 100 mM HEPES–NaOH, 150 mM NaCl, and 6 M guanidinium hydrochloride (Gu-HCl), pH 8.3. Atto647N NHS ester dye was added at a ratio of 1:1 dye:lysines for bovine serum albumin (BSA), ovalbumin (OA), and carbonic anhydrase (CA) or at a ratio of 2:1 for lysozyme (LYS). NHS ester is reactive against primary and secondary amines, such as the N-terminus of a protein (a-amine) and the side chain of lysine (e-amine). Gu-HCl has a guanidinium group similar to the side chain of arginine, in which the electrons are delocalized among the three nitrogen atoms, which provides chemical stability to this group^49,50^. The proteins were labeled overnight (O/N) at 25 °C with 300 rpm shaking. Subsequently, the proteins were pelleted using TCA precipitation to remove the Gu-HCl and washed by acetone on ice. The labeled protein pellets were dried and re-suspended in 2% SDS, 0.5 mM TCEP in 1× Phosphate Buffer Saline (PBS), for 30 min at 37 °C with shaking at 300 rpm.

Whole-cell protein extracts were prepared from HCT116 cells, grown up to 80% confluency in DMEM media containing 1 mM sodium pyruvate and 10% fetal calf serum in a humidified chamber at 37 °C. The cells were trypsinized, harvested, and washed 3 times with PBS. 2 × 10^6^ cells were re-suspended in either Lys or Cys labeling buffers consisting of 100 mM HEPES–NaOH, 150 mM NaCl and 6 M Gu-HCl at pH 8.5 or 7.3, respectively. The cell suspensions were sonicated to further disrupt the cell membrane and induce cell lysis. The lysates were clarified at 20,000*g* for 30 min at 4 °C. Half of the lysate volume (lysate of 10^6^ cells) was used for each labeling reaction. Each lysate was diluted 1:1 in its appropriate buffer, the Cys labeling buffer was adjusted with 40 mM TCEP, and the lysates were incubated for 1 h at 37 °C. Then each lysate was subjected to incubation O/N at 25 °C without dye, with 1 mg of atto647N NHS ester (lysine labeling) or with 1 mg of atto647N maleimide (cysteine labeling). Subsequently, the labeled lysates were precipitated using TCA precipitation followed by four washes with ice-cold acetone. The protein pellets were dried and re-suspended in 2% SDS, 0.5 mM TCEP in PBS shaken at 1,000 rpm for 30 min. at 37 °C.

Samples of labeled recombinant proteins or whole-cell extracts were either subjected to SDS-PAGE analysis followed by fluorescence imaging using the laser gel scanner (Pharos, BioRad) and subsequent Coomassie blue staining or subjected to single-molecule analysis (see Figures S2 and S6). Prior to the latter, the proteins were denatured in 25 mM Tris HCl, 5 mM TCEP, and 2% w/v SDS at 90 °C for 5 min. Proteins were subsequently diluted in running buffer (25 mM Tris HCl, 0.25 M glycine, and 0.1% w/v SDS) to 0.1–0.4 nM.

### Chip fabrication

The chip fabrication followed the description by Zrehen et al.^35^ In short, low-stress SiN_x_ (200 nm) was deposited onto a starting SiN_x_/SiO_2_/Si wafer (50 nm/350 nm/350 µm) by PECVD (300 °C), in order to increase the workable channel depth. Following cleaning in solvents (acetone, methanol, isopropanol, water), the wafer was baked on a hot plate at 300 °C for 10 min. AZ1518 resist was spin-coated at 4,000 RPM to a thickness of ~ 1.8 µm and baked on a hotplate at 120 °C for 2 min. The microchannels were exposed by UV light (MicroWriter ML3), developed in Novo Developer (2.14% TMAH in water), and subsequently etched by reactive ion etching (RIE) with 10 sccm CF_4_ and 10 sccm O_2_ (75 W, 0.15 mbar). Next, 0.5 mm through-ports were exposed as individual squares backside-aligned to the microchannels. Along with cutlines, the SiN_x_ and underlying SiO_2_ insulating layer were etched by RIE and BOE, respectively, and then opened by anisotropic etching in 33% KOH for individual 10 × 10 mm^2^ chips.

### Channel coating

Channels were cleaned by an initial flush with 1 M NaOH for 10 min followed by flushing with deionized (DI) water. Subsequently, the channels were conditioned for acrylamide coating by incubating with 2:3:5 mixture of 3-(trimethoxysilyl)propyl methacrylate, glacial acetic acid, and DI water for 30 min, and then rinsing with methanol and DI water for 10 min each. A solution of 5% (w/v) acrylamide coating containing 5 mg/mL 2,2′-azobis(2-methylpropionamide)dihydrochloride (V-50) photoinitiator was flushed through the channels, and the channels were then exposed to 365 nm UV-light in close proximity (< 3 mm) for 10 min (Spectroline ENB-260C).

### Kymographs construction

The kymographs were constructed by concatenating the central pixel column of each frame (column 256) from a fixed location along the channel—1.3 mm for the 1.5 mm gel and 2.3 mm for the 2.5 mm gel. The pixel values represent the intensity value obtained from the EMCCD camera. The dynamic range of the image is then adjusted to produce a clear image of the labeled proteins.

### Protein detection algorithm

The original accumulated image (before rescaling pixel values) is filtered using a Laplacian of Gaussian (LoG) filter, which results in image segmentation where each segment represents a single protein. The segment center of gravity corresponds to the protein location in the image, and the total energy (pixel value and area) correlates with the protein intensity. The protein’s time of arrival is determined by the center of gravity location along the horizontal axis divided by the frame rate. The protein’s intensity is the normalized value of the segment energy.

## Supplementary information

Supplementary Information 1.

Supplementary Video S1.

Supplementary Video S2.

Supplementary Video S3.

Supplementary Video S4.
