# Supplementary material for: On-chip protein separation with single-molecule resolution
Source: Sci Rep. 2020 Sep 17;10:15313. doi: 10.1038/s41598-020-72463-z (PMC7498591; doi:10.1038/s41598-020-72463-z)
Supplement: Supplementary file 1 — Supplementary Information 1. [file 41598_2020_72463_MOESM1_ESM.pdf]

On-Chip Protein Separation with Single-Molecule Resolution

Supporting Information

Adam Zrehen,[#] Shilo Ohayon,[#] Diana Huttner and Amit Meller*

Department of Biomedical Engineering

Technion - IIT

[#]equal contribution

*Corresponding author. E-mail: ameller@technion.ac.il

Table of Contents:

Video S1-S4 captions

Figure S1- Virtual projection of exposure area

Figure S2- SDS PAGE analysis of Atto647N-labeled recombinant protein

Figure S3- Protein stacking at gel interface

Figure S4- Lysozyme separation

Figure S5- Proteome size analysis

Figure S6- SDS-PAGE analysis of Atto647N-lysine- or cysteine-labeled whole cell extracts

Figure S7- Single protein analysis histogram of Atto647N-lysine-labeled whole cell extracts

Movie captions

*All movies were acquired at a frame rate of 19.31 frames per second and field-of-view of ~ 136 X 136 μm.

Movie S1. Protein sample loading. The movie shows the protein sample flows from the loading port toward the vacuum port due to the negative pressure gradient. The negative pressure is then turned off, equalizing the pressure between the two ports, which stops the sample flow. The next step is applying a positive potential between the ground port (left) and the positive electrode port (right). The proteins move in the direction of the electric field towards the positive potential located at the right port (positive electrode) and bottom port (due to leakage through the silicon). The distinct feature of the electrical field allows only a finite amount of proteins to enter the gel and prevent protein leakage from the loading port, as can be seen.

Movie S2. Protein stacking at the gel interface. The movie shows a controlled amount of proteins stacking at the gel interface. The plug of proteins arrives at high velocity, and as individual proteins enter the gel, they slow down, reducing the width of the plug over time.

Movie S3. Protein band crossing the gel. The movie shows a protein band crossing the gel at a position of 1.3 mm from the gel interface. Each protein in the band can be easily distinguished to allow single-molecule analysis. The protein band shows a normal distribution of the protein's density across the channel, as expected.

Movie S4. Proteins exit the gel. The movie shows proteins leaving the gel medium back to the buffer. As they do so, the protein's velocity increases due to the high mobility in the buffer compared to the mobility in the gel.

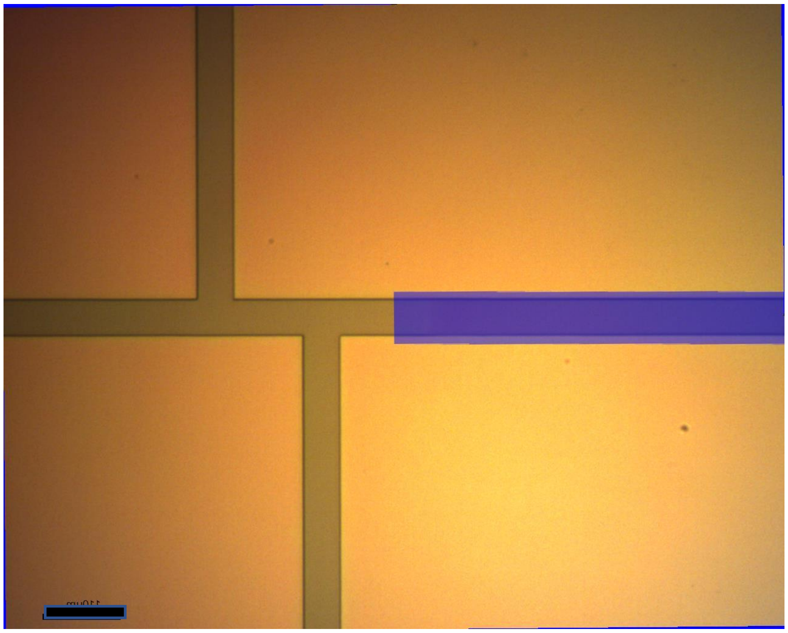

Figure S1. Virtual projection of exposure area. The purple rectangle overlaid atop the separating channel indicates the area to be exposed by UV-light using the MicroWriter ML3 (DMO) direct-write photolithography machine (365 nm, ~12500 mJ/cm^2 or ~5 mins). Exposure extends outside the field-of-view, down the length of the separating channel. The maximum write-field size is ~1.55 mm x 1.55 mm using a 3x objective. Bar corresponds to 100 µm.

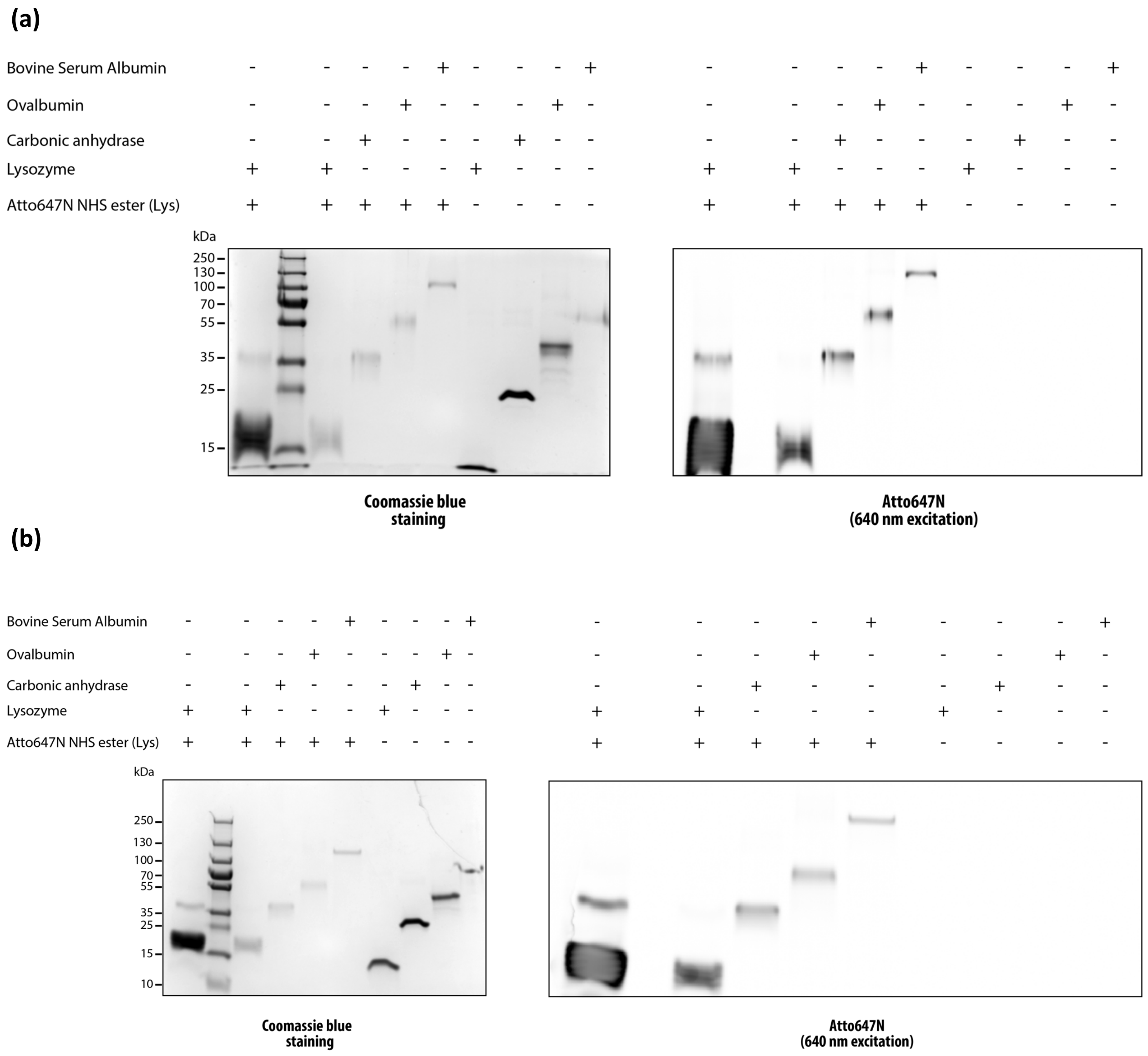

Figure S2. SDS PAGE analysis of Atto647N-labeled recombinant proteins. Proteins were labeled on their lysine residues as described in the Materials and Methods section in the main text and subjected to separation either on a **(a)** 12% gel, or on a **(b)** 4-20% gradient gel. Atto647N fluorescence was imaged using the laser gel scanner (Pharos, BioRad) and a 640 nm excitation, right panels. Subsequently, the gels were stained with Instant Coomassie (Expedeon), thoroughly destained in water, and imaged using the Gel imager (GelDoc, BioRad), left panels. In all cases, the atto647N-lysine-labeled proteins show a retarded migration as compared with their unlabeled counterparts.

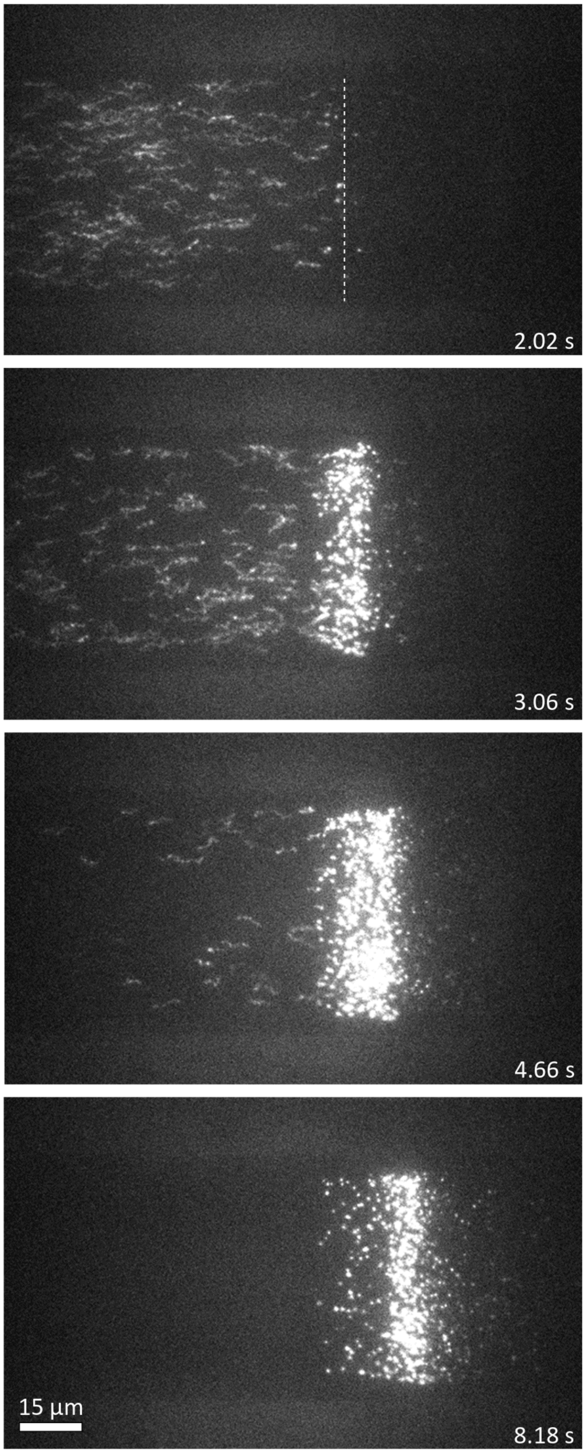

Figure S3. Protein stacking at gel interface. Atto647N-labeled BSA proteins are driven towards the polyacrylamide gel at a 30 V applied bias (the interface of the gel is indicated by the white dashed line). The initial plug with an end-to-end length of ~200 µm is concentrated to a width of ~10 µm at the gel interface by t= 8.18 s. The 8% gel was exposed by 25,000 mJ/cm^2.

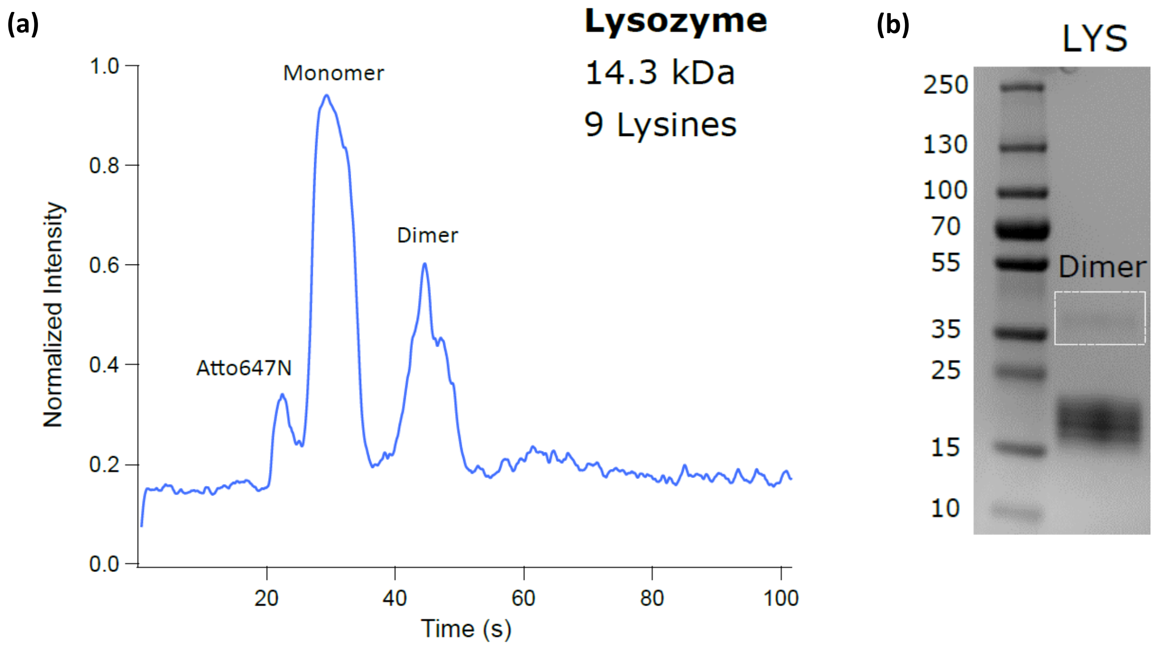

Figure S4. Lysozyme separation. (a) Atto647N-labeled lysozyme proteins were separated into dimers and monomers in a polyacrylamide gel (8%, 12,500 mJ/cm^2) at 30 V applied bias. The three peaks corresponding to the Atto647N free fluorophores, monomers, and dimers, are labeled. Lysozyme contains 9 lysine residues, and the monomer weighs 14.3 kDa. **(b)** Lysozyme separated on a 4-20% gel showing the atto647N fluorescence of the monomer and dimer bands.

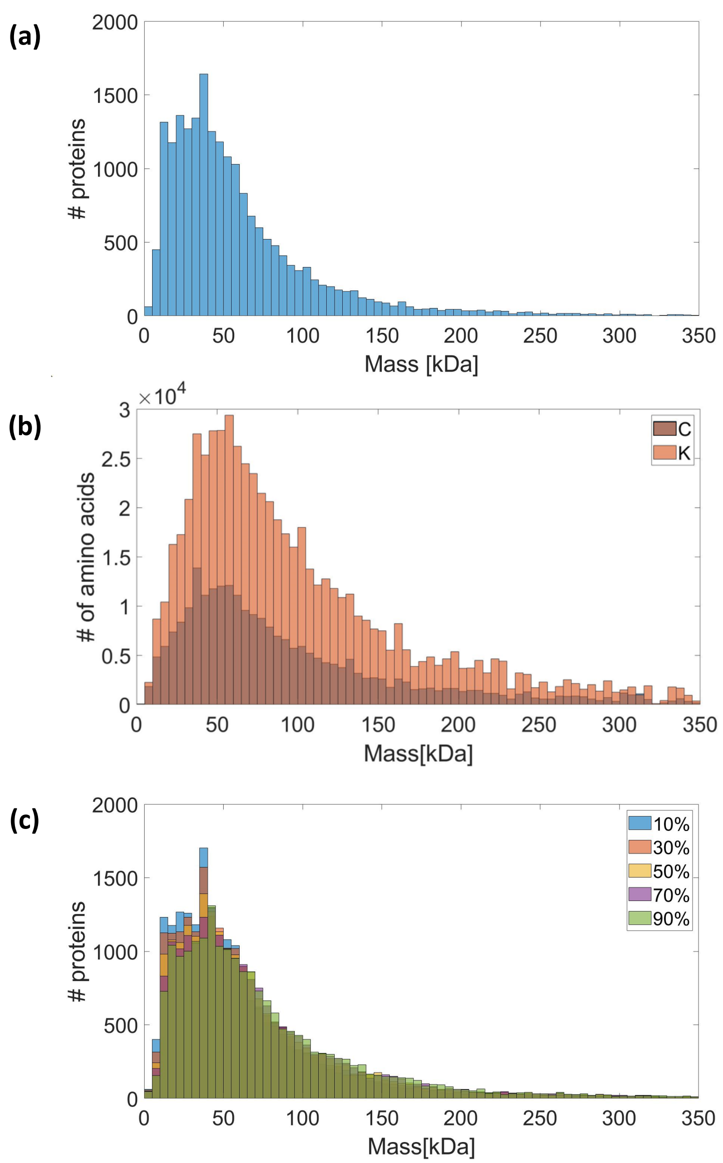

Figure S5. Proteome size analysis. Whole proteome analysis of the human proteome according to the SwissProt database. The histograms present the protein size distribution with a bin size of 5 kDa (5000 Da) in the range 0 to 350 kDa. The proteins accounted for in this range include 99.34% of the whole proteome. The remaining 0.66% (rare proteins of molecular weight > 350 kDa) were excluded in order to simplify the presentation. (a) Histogram of the number of proteins according to their mass distribution. (b) Histogram of the number of cysteines (C) and lysines (K) distribution in the human proteome according to protein mass. (c) Histogram of the number of proteins as a function of their mass distribution, taking into account certain degrees of lysine (K) labeling efficiencies (10%, 30%, 50%, 70%, 90%) as indicated. For each protein in the proteome the number of K-labels was determined by the number of K in the protein times the labeling efficiency. Each labeled K adds 628.9 Da (MW of conjugated Atto647N) to the protein's total mass.

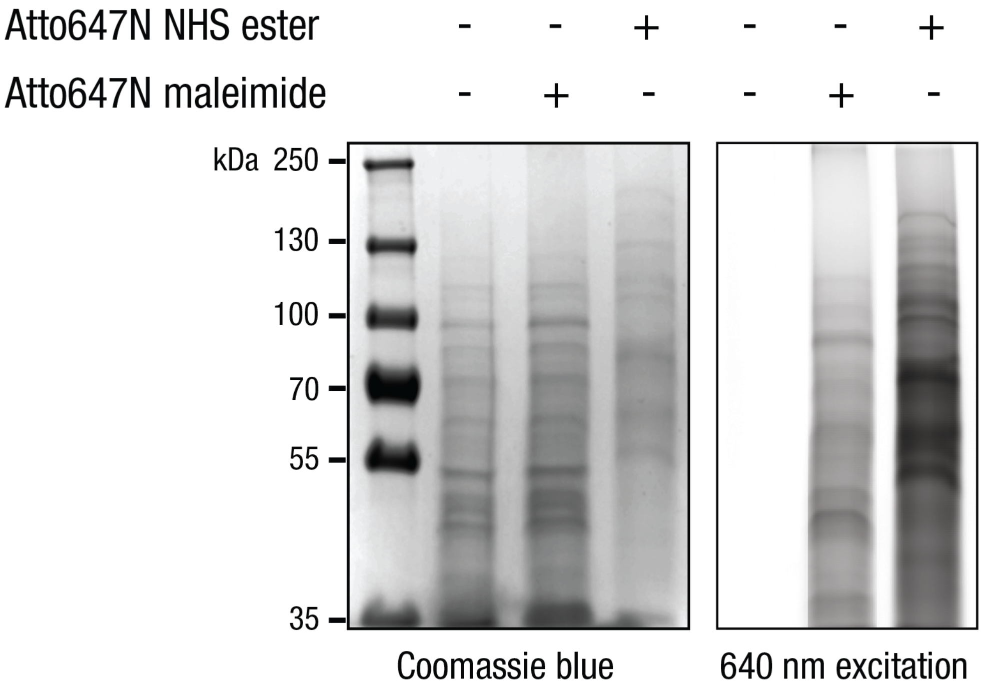

Figure S6. SDS PAGE analysis of Atto647N-lysine or cysteine labeled whole-cell extracts. 1 x 10^6 cells were lysed in a buffer containing 100 mM HEPES-NaOH, 6M Gu-HCl and 150 mM NaCl buffer, either at pH 8.5 or at pH 7.3, corresponding to either Lysine or Cysteine labeling, accordingly. The lysates were clarified at 20,000g, 4°C for 30 min, and then diluted 1:1, each in its appropriate buffer, adjusting the lysate used for cysteine labeling with 40 mM TCEP. Each lysate was incubated for 1 h at 37°C and subjected to incubation O/N at 25°C either with DMSO (no dye), with atto647N NHS ester (for lysine labeling) or with atto647N maleimide (for cysteine labeling). Subsequently, the labeled proteins were precipitated using TCA precipitation followed by 4 washes with ice-cold acetone. The protein pellets were dried and resuspended in resuspension buffer consisting of 2% SDS, 0.5 mM TCEP in PBS and dissolved at 1000 rpm for 30 min at 37°C. The samples were separated on an 8% SDS-PAGE gel, and the labeled proteins were visualized using laser gel scanner (Pharos, BioRad). Subsequently, the gel was stained with Coomassie blue and imaged using gel imager (GelDoc, BioRad). In each lane, the amount of protein lysate, which was used, is indicated. For better visualization of fluorescence, different ratios of labeled lysates were loaded, as indicated.

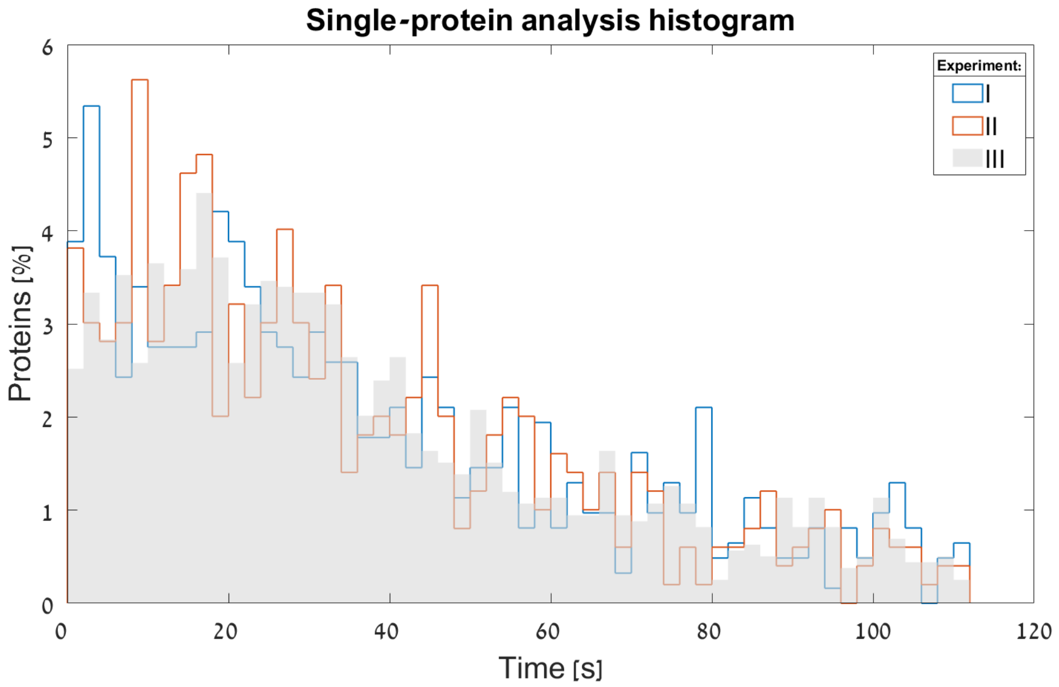

Figure S7. Single protein analysis histogram of Atto647N-lysine labeled whole-cell extracts. Three whole-cell extract separation experiments (I-III) preformed using three different devices. TNormalized histograms present single-protein identification as a function of migration time (referenced to fluorophore front, and bin size of 5 seconds). The overall shape of the histograms shows a similar trend with distinct peaks. The variation between the experiment peaks can be explained by the use of different devices of slightly different dimensions and gel properties, and slight variation in the identification of the flurophore peak front causing a systematic error.
